# Translationally Controlled Tumor Protein, a Dual Functional Protein Involved in the Immune Response of the Silkworm, *Bombyx mori*

**DOI:** 10.1371/journal.pone.0069284

**Published:** 2013-07-22

**Authors:** Fei Wang, Cuimei Hu, Xiaoting Hua, Liang Song, Qingyou Xia

**Affiliations:** State Key Laboratory of Silkworm Genome Biology, Southwest University, Chongqing, China; Uppsala University, Sweden

## Abstract

Insect gut immunity is the first line of defense against oral infection. Although a few immune-related molecules in insect intestine has been identified by genomics or proteomics approach with comparison to well-studied tissues, such as hemolymph or fat body, our knowledge about the molecular mechanism underlying the gut immunity which would involve a variety of unidentified molecules is still limited. To uncover additional molecules that might take part in pathogen recognition, signal transduction or immune regulation in insect intestine, a T7 phage display cDNA library of the silkworm midgut is constructed. By use of different ligands for biopanning, Translationally Controlled Tumor Protein (TCTP) has been selected. BmTCTP is produced in intestinal epithelial cells and released into the gut lumen. The protein level of BmTCTP increases at the early time points during oral microbial infection and declines afterwards. *In vitro* binding assay confirms its activity as a multi-ligand binding molecule and it can further function as an opsonin that promotes the phagocytosis of microorganisms. Moreover, it can induce the production of anti-microbial peptide via a signaling pathway in which ERK is required and a dynamic tyrosine phosphorylation of certain cytoplasmic membrane protein. Taken together, our results characterize BmTCTP as a dual-functional protein involved in both the cellular and the humoral immune response of the silkworm, *Bombyx mori*.

## Introduction

Sensing or recognition of invading microorganisms is the crucial step to trigger effective immune response in animals and plants [1], [2]. Distinct pathogen-associated molecular patterns (PAMP) that different microorganisms possess, such as lipopolysaccharide (LPS) or peptidoglycan (PG), are detected by membrane-bound or circulating molecules in hosts, which are often referred to as pattern recognition receptors (PRRs). Although finer discriminations between pathogenic and non-pathogenic microorganisms, or between infectious or commensal colonies, even between viable or dead pathogens can not be fully explained by the interaction between these receptors and their ligands, studies in different metazoa have revealed that they are evolutionarily conservative and constitute an essential part of innate immunity.

Insect humoral and cellular immune response is considered to be initiated through distinct receptors [3], [4]. The two canonical signaling pathways that would lead to the massive production of anti-microbial peptides (AMPs), Toll and IMD pathways are triggered by different types of receptors which are categorized according to the disparate bacterial cell wall components they recognize, such as peptidoglycan recognition protein (PGRP) and β-1,3-glucan recognition protein (βGRP). Whereas, pathogen recognition followed by internalization generally requires the transmembrane phagocytosis receptors, such as the scavenger receptors, the Ig-SF-domain containing protein isoforms, Down syndrome cell adhesion molecules (Dscams), or the epidermal growth factor-containing protein Eater to complete the uptake of pathogens [5]–[9]. In addition, some opsonic molecules, such as thioester-containing proteins (TEPs) facilitate phagocytosis of microbes by forming a complex with them which is plausibly recognized by certain transmembrane receptors [10], [11].

Besides the systematic immune response which has been intensely studied in hemocytes and fat body, local anti-microbial reaction that happens in epithelial tissues also makes a great contribution to the defense against pathogen infection [12]. Actually, intestinal epithelia deter microbes and act as the first line of defense in natural circumstances since many pathogens are likely introduced via feeding. Although several studies report generation of ROS and production of AMP are key to controlling infection in the gut, [13]–[15], a comprehensive delineation of the interactions between intestinal epithelium and invading microorganisms is required for better understanding of the intestinal immunity. Currently, discovery of microorganism sensing molecules expressed in insect intestine mainly relies on transcriptomic or proteomic analysis with comparison to the similar molecules that have been identified to be PRRs in other tissues [16], [17]. However, due to the difference in protein abundance and susceptibility to detection between intestine and other tissues, only a few PRRs have been identified in the insect intestine.

In this study, we have taken an alternative approach by constructing a phage display cDNA library of the silkworm midgut and carrying out high throughput screening of pathogen binding molecules. Phage display technology has already been successfully applied in selection of various bio-reactive peptides targeting specific molecules, or even cells [18], [19]. cDNA library is constructed in bacteriophage T7 which enables surface display of silkworm midgut proteins with no privilege of their different features. Individual phage with a unique protein displayed on its surface that interacts with the bait, which would be different pathogenic bacteria or PAMPs in this study, was isolated and amplified. Out of the resulting peptides/proteins, some of which have already been characterized to be PRRs by previous reports, we found one molecule, annotated as Translationally Controlled Tumor Protein (TCTP), displays a broad range of pathogen-binding capacity. TCTP has been characterized as a multifunctional protein by different studies. It promotes allergic response in mammalian cells by inducing the release of histamine from basophiles or mast cells [20]. It is also involved in microtubule-stabilization, anti-apoptosis and embryogenesis [21], [22]. A few studies in invertebrates suggest TCTP might act as an anti-bacterial or anti-viral factor. Knock-down of *TCTP* in *Drosophila melanogaster* by RNAi decreased the viability upon oral infection of *Serratia marcescens* [23]. Inoculation of bacteria, virus or parasitical wasp resulted in an increased expression level of *TCTP* in *Penaeus monodon*, *Parribacus japonicus* or *Plutella xylostella* [24]–[26]. Particularly, when *P. japonicus* was infected with White Spot Syndrome Virus (WSSV), potent expression of *TCTP* along with other immune-related genes, including C-type lectin and interferon-like protein, was detected [27]. In addition, injection of purified TCTP into WSSV infected *P. monodon* increased the survival rate of *P. monodon* and suppressed the proliferation of WSSV. The anti-viral activity was also confirmed in Sf9 cells [28]. In spite of these findings, no clear picture of the overall immunological role of TCTP in invertebrates has emerged. In this study, the pathogen-binding properties of BmTCTP were characterized, and the cellular and humoral immune response it might participate was also investigated.

## Materials and Methods

### Animals, Bacteria and Cell Lines

Silkworm larvae (DaZao P50 strain) were reared with fresh mulberry leaves at 25°C and relative humidity of 80%. *Bacillus bombyseptieu*s was kindly provided by Professor Yanwen Wang (Silkworm Diseases Laboratory of Shandong Agriculture University, China). *Serratia marcescens*, *Bacillus thuringiensis*, *Escherichia coli* and *Staphylococcus aureus* were maintained in our lab. BmNSWU1 cells (BmNs) were established from the ovarian tissues of silkworm larvae in our lab [29] and cultured in TC-100 medium with 10% fetal calf serum at 27°C.

### Construction of a T7 Phage Display cDNA Library of the Silkworm Midgut and Panning of Ligand-binding Peptides from T7 Phage Display Library

The midguts were freshly dissected from the fifth instar silkworm larvae. Total RNA was extracted by Trizol (Life Technologies, USA) according to the manufacturer’s instructions. The RNA samples were further treated with DNase I (RNase free) to remove any DNA contamination. 2 µg of total RNA was reverse-transcribed with a First-Strand cDNA Synthesis Kit (Promega, USA). Double-stranded cDNA was synthesized by use of T7 select 10-3 OrientExpress cDNA Cloning System, Oligo (dT) kit (Novagen, Germany) and then cDNA ends were modified to ligate with directional *EcoRI/HindIII* linkers. The purified cDNA was ligated into the T7 select vector, packaged into phage and amplified to generate the library for biopanning. Finally, phage titers were determined by plaque assays according to the manufacturer’s instruction. For biopanning, T7 phage display cDNA library of the silkworm’s midgut was applied to different ligand, including chitin, lipopolysaccharide (LPS), *B. bombyseptieus* and *S. marcescens*. In each panning round, unbound phages were removed by intensive washing. Remaining bound phages were eluted and allowed to infect *E. coli* (BLT5403) to amplify. Then a new round of panning was performed with the amplified phage population. To identify the peptides that selected by ligands, PCR amplification was performed on randomly chosen plaques and the amplified fragments were cloned into pMD19-T simple vector and confirmed by DNA sequencing. BLAST algorithm was used to determine their identity by homologous alignments to NCBI (http://www.ncbi.nlm.nih.gov/BLAST) and SilkDB (http://www.silkdb.org/silkdb/).

### Prokaryotic Expression and Purification of Recombinant Proteins

TCTP-coding sequence (GI:112982879) was cloned into the pET28a expression vector with 6×His tag at the N-terminus. The recombinant plasmids were transformed into competent BL21 (DE3) strain. Bacteria were collected after induced with 0.2 mM IPTG at 37°C for 4 h and then treated by ultrasonic disruption. The recombinant protein was purified by Ni^2+^ affinity chromatography and dialyzed overnight in PBS (20 mM Na_2_HPO_4_, 20 mM NaH_2_PO_4_, pH 7.2). Finally, the purified protein was used to immunize rabbits to generate polyclonal antibody (Genscript, China). The contaminated endotoxin was removed from the recombinant protein using ToxinEraser™ endotoxin removal resin (Genscript). For cell assays, only endotoxin-removed recombinant protein was used.

### Immunohistochemical Analysis of the Intestinal Expression of BmTCTP

Immunostaining was performed in 4 µm sections of neutral-buffered formaldehyde-fixed paraffin-embedded midgut tissues. All sections were routinely mounted on slides and dried at 60°C for 30 min. Antigen retrieval was carried out by microwave heating in sodium citrate buffer pH 9.0 at 100°C for 20 min. Following blocked with 5% goat serum at 37°C for 1 h, sections were incubated overnight (4°C) with the rabbit anti-BmTCTP antibody (1∶100) and rinsed with PBS. A goat anti-rabbit FITC-labeled antibody (1∶500) (Beyotime, China) was employed as the secondary antibody followed with a brief nucleus staining with DAPI (Beyotime). Finally, the sections were covered with coverslips and sealed with anti-fading mounting medium. Pre-immune rabbit serum was used as the negative control.

### Binding of BmTCTP to Bacterial Cells and Chitin

Single colonies of *B. bombyseptieus*, *S. marcescens*, *B. thuringiensis* and *E. coli* were individually grown in 4.0 ml LB medium at 37°C at 220 rpm until OD600 was close to 1.0. After centrifugation at 4,000 g and washing twice with 20 mM PBS, cells were resuspended in PBS. Purified BmTCTP (5 µg) in 200 µl PBS was mixed with the pellet from 2 ml cell suspension at 4°C for 2 h. After centrifugation at 4,000 g for 10 min, the cell pellet was washed four times and resuspended in 40 µl PBS. Following separation on 15% SDS-PAGE gel and electro-transfer, the protein bound on cell pellet and the last wash was detected by immune blot using anti-His monoclonal antibody (Beyotime). Similarly, chitin (2 mg) and 5 µg BmTCTP were incubated at 4°C for 2 h. After 10,000 g centrifugation and washing four times with PBS, the pellet was preceded as described above.

### Plate Assay of BmTCTP Binding to Peptidoglycans and Lipopolysaccharide

Peptidoglycans (PG) of *B. subtilis* and lipopolysaccharide (LPS) of *E. coli* (Sigma-Aldrich, USA) were applied to wells (2 µg/well) of a 96-well microplate, air dried overnight at room temperature, and fixed to the wells at 60°C for 30 min. The wells were blocked with 200 µl of 1 mg/ml bovine serum albumin (BSA) in Tris buffer saline (150 mM NaCl, 20 mM Tris HCl, 0.05% Tween 20) at 37°C for 2 h and washed with 200 µl TBST for five times by microplate washer (Bio-Rad). BmTCTP (100 ng) in 100 µl PBS containing 0.1 mg/ml BSA was added to the wells and incubated at room temperature for 3 h. Following the washing step with TBST, 100 µl of 1∶1,000 diluted anti-BmTCTP antibody in TBST containing 0.1 mg/ml BSA was added to the wells and incubated at 37°C for 2 h. After washing with 200 µl TBST for five times, 100 µl 1∶3,000 diluted goat anti-rabbit IgG conjugated to alkaline phosphatase in TBST containing 0.1 mg/ml BSA was added to the wells and incubated at 37°C for 2 h. After washing with TBST for five times, 3,3′,5,5′-Tetramethylbenzidine was added to the wells (100 µl/well) and incubated for 15 min at 37°C shielded from light. 100 µl 1 mol/L H_2_SO_4_ was added per well to stop the reaction and the absorbance at 450 nm was monitored in the microplate reader (Promega).

### Phagocytosis Assays

Red fluorescence labeled latex beads (5×10^8^) (Sigma) were suspended in 50 µl BmTCTP (0.2 µg/µl) solution and incubated at 25°C for 1 h. Then the mixture was injected into 5 larvae (day 2 of 5^th^ instar, 10 µl/larvae) using a capillary glass tube, and the hemolymph was collected via pricking abdominal foot of larvae with sterilized needle 1 h later. Collected hemolymph was immediately diluted with ice-cold N–phenylthiourea-saturated PBS in a 2 ml centrifuge tube. The coverslips were put in 12-well cell culture plate in advance and the silkworm hemolymph was dropped on them gently. After cells adhered to the coverslips, they were fixed with 4% paraformaldehyde for 15 min. Then cells were washed three times with PBS and stained with green fluorescent probe (DiO) (Beyotime) for 20 min followed by three washes. Finally nucleus was stained with DAPI. In the end, coverslips were taken out from the wells and mounted on the microscope slides. Cells were visualized under the fluorescence microscope (Nikon ECLIPSE 80i, Japan). The fluorescence-labeled particles co-localized with hemocytes were counted in 10 microscopic fields for each sample. BSA was used as the control under similar conditions described above. For phagocytosis assay of *S. aureus* or *S. marcescens*, the same procedure was followed except bacteria were first labeled with FITC (Sigma-Aldrich) according to a standard procedure. Briefly, 1×10^6^ bacteria were harvested and suspended in 0.1 M carbonate buffer (pH 9.5) containing FITC (1 mg/ml). The mixture was incubated for 2 h at room temperature. Then the bacteria were collected by centrifugation, washed three times with PBS, and resuspended in carbonate buffer.

### Silkworm Intestinal Infection and Extraction of Gut Lumen Soluble Protein

Mulberry leaves were disinfected with bleaching powder and painted with bacterial suspension. 1×10^10^ *B. bombyseptieus* or *S.* *marcescens* were fed to each silkworm larva and at least ten larvae were used for different bacterial infection. Then the gut lumen protein was prepared as described by Pauchet *et al.* [30]. Briefly, midgut was dissected from the third day fifth-instar larvae in ice-cold PBS. Peritrophic matrix containing the food bolus was pulled out of the midgut and the remained tissue was gently homogenized by 10 strokes in a homogenizer in PBS, pH 7.5, containing a mixture of protease inhibitors to release soluble proteins. After centrifugation (13,000 g, 1 h, 4°C), the supernatant containing the gut lumen soluble proteins were collected and protein concentration was determined using the BCA method. Gut juice was collected from the regurgitates that silkworm larvae expelled elicited by chloroform.

### RNA Interference (RNAi) and Quantitative RT-PCR (qRT-PCR) Analysis

Template for dsRNA synthesis was generated by PCR using primer pair specific for ERK (GI:112982893): forward 5′-TAATACGACTCACTATAGGGAGAGAACATCAAACGTATTGCCAG AG-3′ and reverse 5′-TAATACGACTCACTATAGGGAGACGAACCGAGCACTCCCAAA-3′. Then dsRNA was *in vitro* synthesized using T7 RiboMAX Large Scale RNA Production System (Promega) following the manufacturer’s instruction. BmNs cells were seeded at 1×10^5^ per well in a 12-well culture plate and transfected with 5 µg dsRNA using X-treme GENE Transfection Reagent (Roche, Switzerland). Before induction cells were changed into serum-free medium, and then the endotoxin-removed recombinant BmTCTP was applied with or without chemical U0126 or PD098059 (Cell Signaling Technology). Total RNA was extracted from cells using Total RNA Kit (Omega) at different time points and qRT-PCR was performed using SYBR Premix Ex Taq II (TaKaRa, Japan) on a StepOne Plus Real-Time PCR System (Applied Biosystems, USA) with a program consisting of an initial denaturing step of 30 s at 95°C and 40 amplification cycles consisting of 5 s at 95°C followed by 30 s at 60°C. The expression level of anti-microbial peptides was normalized to the control (SilkDB Probe number: sw22934).

### Immunoblotting of Cytosolic or Plasma Membrane Protein

Cytosolic and cell membrane protein was extracted from BmNs cells using the same methods described by Wang *et al.* [31]. Protein concentration was estimated by BCA assay and 20 µg protein per sample was resolved on 12% SDS-PAGE. After transferred to PVDF membrane (GE Health Care, USA), cytosolic samples were immunoblotted with anti-ERK polyclonal antibody (Cell Signaling Technology) or anti-Tubulin mAb (Sigma-Aldrich), and cell membrane samples were immunoblotted with anti-phosphotyrosine mAb 4G10 (Cell Signaling Technology) following the standard procedure.

### Statistical Analysis

The experiments were repeated three times with similar results. All data from the analysis of relative levels or cell counts were expressed as means ± standard deviation (S.D.). The statistical significance of differences was determined using Student’s *t* test.

## Results

### Selection of Ligand-binding Peptides from the Silkworm Midgut by Biopanning

In order to screen the ligand-binding molecules from the silkworm midgut, we generated a T7 phage display cDNA library for biopanning. The size of inserts in phage ranged from 0.3 to 2.0 kb examined by PCR of randomly selected plaques. The primary library titer was 1×10^5^ pfu/ml, and after amplification the titer was 8.0×10^10^ pfu/ml.

Pathogen-associated molecules, including LPS which has been immobilized on microtiter plate wells and chitin, or pathogenic microbes to silkworm, including *B. bombyseptieus* and *S. marcescens* were used as the ligands for selection of the T7 phage display cDNA library. Typically five rounds of panning were performed to ensure no further increases in recovered phage number. After final amplification, 30 to 70 plaques were randomly picked and the inserts which encode the peptides displayed on phage were sequenced. The deduced amino acid sequence of peptides was subjected to BLAST analysis and the identity of in-frame sequence is sorted by the ligands that picked it up (Table 1).

**Table 1:** Partial list of in-frame hits identified from the silkworm midgut using phage display.

Protein Identity (SilkDB No.)	Chitin	LPS	Bb	Sm
**Translationally controlled tumor protein (BGIBMGA003073)**	✓	✓	✓	✓
β-glucan recognition protein (BGIBMGA000353)		✓		
Peritrophic membrane chitin binding protein (BGIBMGA013756)	✓			✓
Aminopeptidase N (BGIBMGA008060)	✓		✓	
acyl-CoA binding protein (BGIBMGA002905)		✓		✓
Fungal protease inhibitor F (BGIBMGA009094)		✓		
Hsp70 (BGIBMGA010635)		✓		✓

Translationally Controlled Tumor Protein in bold. LPS, Lipopolysaccharide; Bb, *Bacillus bombyseptieus*; Sm, *Serratia marcescens*. ✓ indicates the protein was recovered in the biopans when corresponding bait was used.

A diversity of in-frame hits was observed among the biopans selected by the different ligands. For instance, a β-glucan recognition protein, which has already been identified to be a PRR in silkworm [32], only appeared in the biopans recovered from LPS screening. By contrast, a peritrophic membrane chitin binding protein, was picked up by chitin and *S. marcescens*. In addition, an aminopeptidase N protein was obtained by biopanning against chitin as well as *B. bombyseptieus*. This glycosylphosphatidylinositol (GPI)-anchored protein which is located on the microvilli of the midgut has been identified to bind Cry toxin and predicted to mediate the pathogenesis of *B. thurngiensis* in silkworm [33]. Based on the characteristics of these in-frame hits, phage display in this study would be considered to be valid and efficient in discovery of novel protein which has not been identified to be pattern or microbe recognition molecules in other studies.

Surprisingly, TCTP was unanimously picked up by all the ligands. To exclude the possibility of its unspecific binding to any substance, we used BSA-blocked microtiter plate well as control and did not find it in the biopans. We also used another pathogenic microorganism, *Bombyx mori* nuclear polyhedrosis virus (BmNPV) particle as the ligand. Similarly, no TCTP sequence-containing phage was obtained.

### Expression and Secretion of TCTP in the Silkworm Midgut

**Figure 1:**
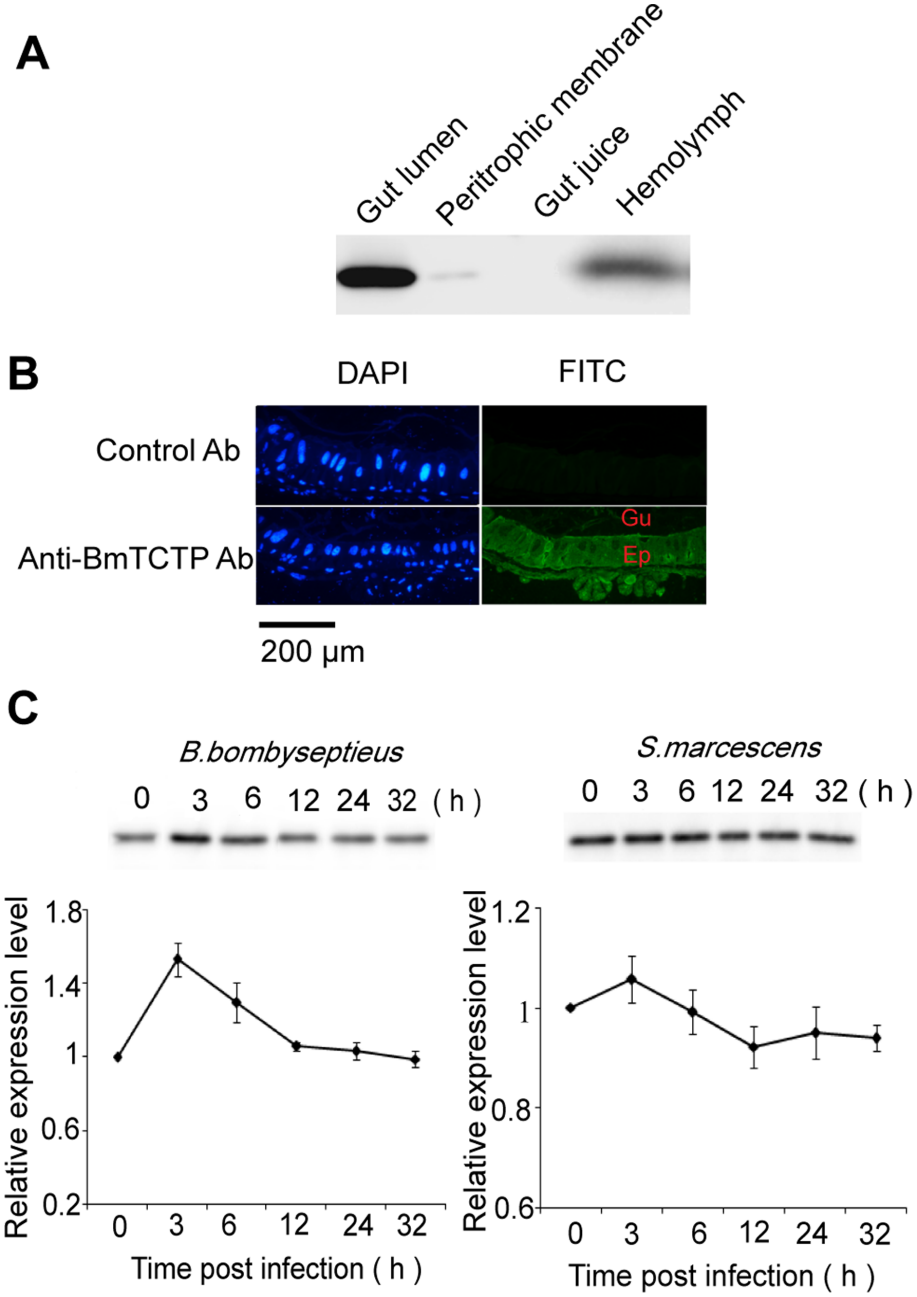
Expression and localization of BmTCTP in the silkworm midgut. A. The existence of BmTCTP in midgut lumen, peritrophic membrane, gut juice and hemolymph of the silkworm was analyzed by Western blotting. Equal amount of protein (20 µg) extracted from each tissue was subjected to SDS-PAGE analysis followed with immunoblotting analysis. B. The expression of BmTCTP in midgut epithelial cells was analyzed by immunohistochemistry. The nucleus was stained with DAPI, and BmTCTP was stained with anti-BmTCTP polyclonal antibody followed with FITC-conjugated secondary antibody, rabbit pre-immune serum was used as negative control. Gu, gut lumen; Ep, epithelia. C. The expression level of BmTCTP in midgut lumen after oral infection of *B. bombyseptieus* or *S. marcescens* at indicated time point was examined by Western blotting, then normalized to the amount of BmTCTP level at 0 h and annotated as mean fold increase (± S.D.). Experiment was repeated three times and representative images were shown here.

Although TCTP, which is highly conserved among diverse taxonomic species, does not contain a signal-peptide sequence, the existence of this molecule in various biological fluids or extracellular spaces has been observed. The mechanism of TCTP secretion has not been clarified, presumably via the exosoma vesicles. In this study, western blotting and immunohistological analysis were performed to determine the intestinal localization of BmTCTP. A high level of BmTCTP was detected in the soluble protein contents of larval midgut and trace amount in the peritrophic membrane, but not in the intestinal juice by western blotting (Figure 1A). We also found the presence of BmTCTP in the hemolymph, but at a much lower level which was approximately 1.0 µg/ml compared to that in gut lumen. Although some intense fluorescence signal was observed in dispersed area, BmTCTP is almost equally distributed inside the intestinal epithelial cells, suggesting that protein detected in gut lumen originates from these massive pools (Figure 1B).

To further investigate whether the expression of BmTCTP in the silkworm midgut was affected during the immune response against microbes, silkworm larvae were fed with *B. bombyseptieus* or *S. marcescens* respectively, and the protein level was examined in the course of infection (Figure 1C). Both bacterial infections induced an increase in the amount of protein secreted into the gut lumen in the earlier time points (3 h). A following decrease was observed, only BmTCTP reduced to a lower level after orally infected with *S. marcescens*.

### TCTP Binds Pathogen-associated Pattern or Bacteria in vitro and Enhances Phagocytosis of Hemocytes

**Figure 2:**
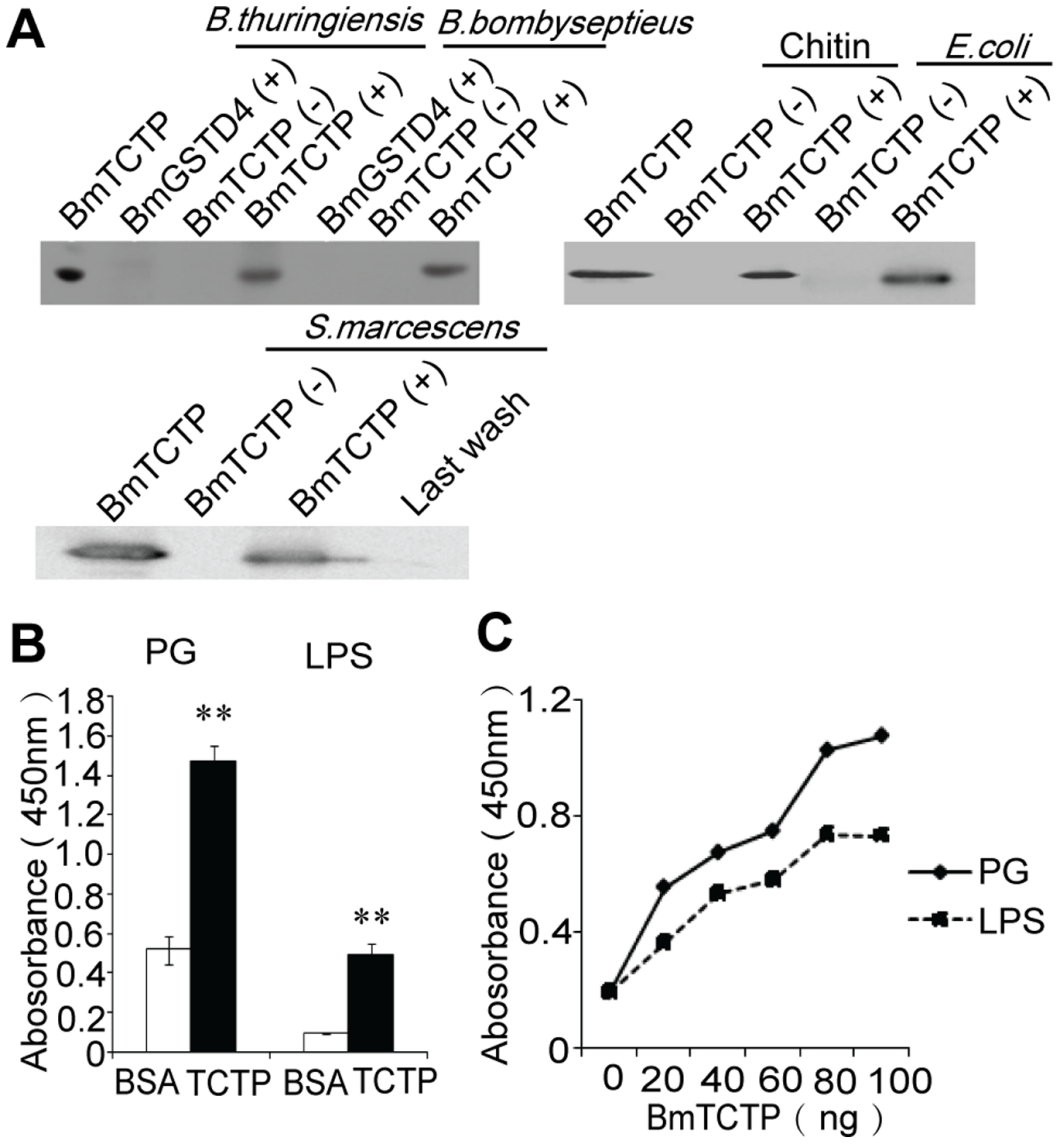
*In vitro* binding assay of BmTCTP to microorganisms and pathogen-associated molecules. A. Recombinant His-tagged BmTCTP bound to various bacteria and chitin were subjected to SDS-PAGE and immunoblotting analysis using anti-His antibody. Recombinant His-tagged BmGSTD4 and the last wash from *S. marcescens* were used as a control. +, with recombinant protein; −, without recombinant protein. B. Association of BmTCTP with soluble PG and LPS which has been immobilized on a 96-well plate was measured by ELISA. 100 ng BmTCTP or BSA was incubated with plate-bound PG and LPS, and the absorbance at 450 nm of each well was compared. C. Increasing amount of BmTCTP was incubated with PG or LPS and detected by ELISA. Values represented by alkaline phosphatase activity are shown here as mean ± S.D. Significant difference between bindings of BmTCTP and BSA to ligands is marked by asterisks (**p<0.01).

In order to confirm the binding capacity of BmTCTP to different ligands, we performed *in vitro* binding assay using His-tagged recombinant BmTCTP. Immuno-blotting by anti-His antibody detected the presence of recombinant BmTCTP from pellets of all bacteria used in this assay, including *B. thuringiensis*, *B. bombyseptieus*, *E.coli* and *S. marcescens* as well as chitin, but no Glutathione S-transferase δ4 of silkworm (BmGSTD4) which was also tagged with 6 × His and used as the control (Figure 2A). To exclude the possibility that BmTCTP unspecific-binding might not be depleted from bacterial pellets by intensive wash, we also checked the last wash and did not find the presence of BmTCTP.

To investigate whether bacterial cell surface components such as PG or LPS contributes to the binding of BmTCTP, we used an ELISA-based binding assay to examine the specific binding of BmTCTP to soluble PAMP (Figure 2B). Although unspecific binding was observed in control, BmTCTP demonstrated significant higher capacity in binding to these ligands, and the bindings occurred in a dose-dependent manner (Figure 2C).

**Figure 3:**
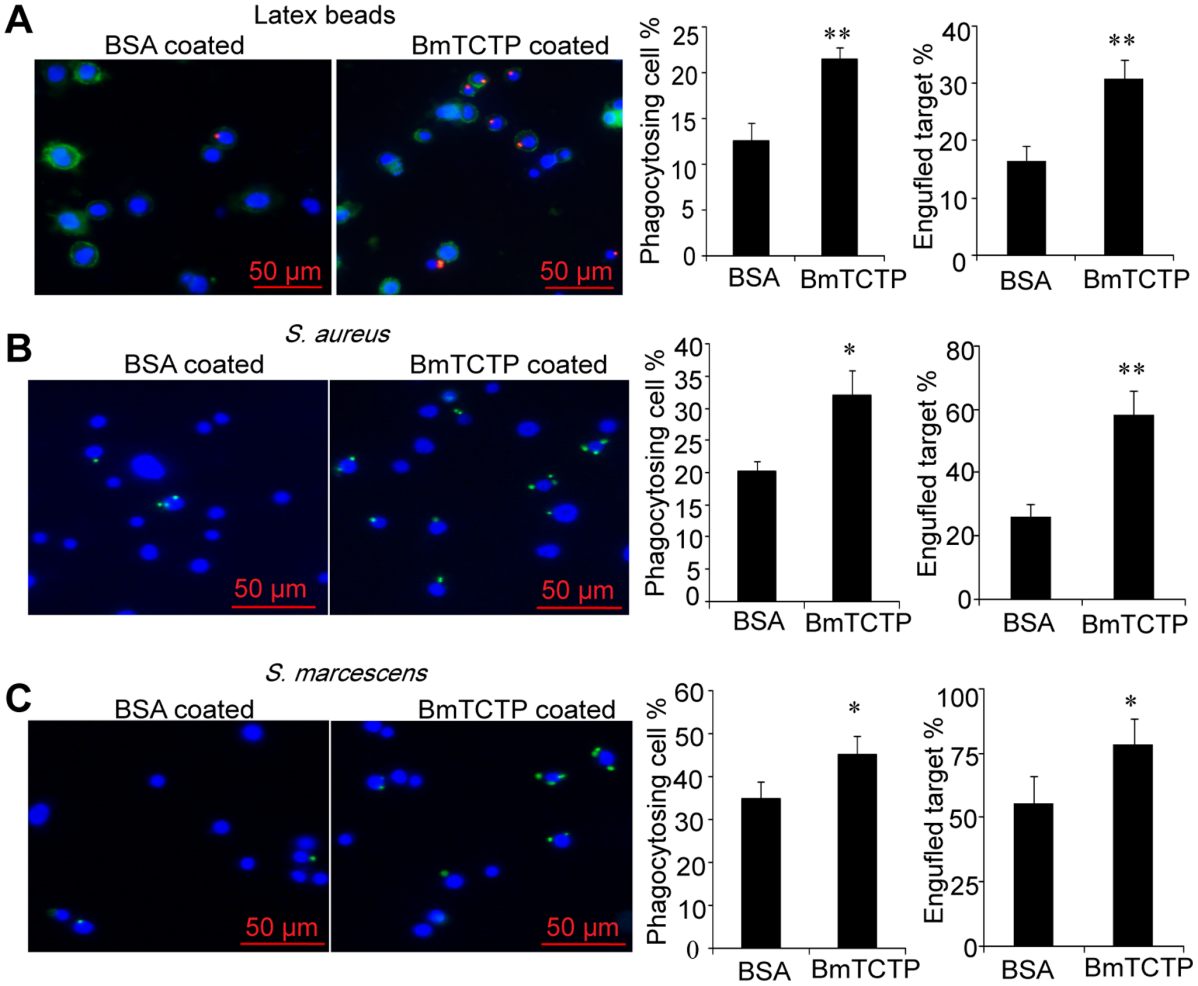
BmTCTP-mediated phagocytosis by silkworm hemoctyes. Internalized red fluorescence-labeled latex beads (A) and FITC-labeled bacteria (B, C) pre-coated by BmTCTP or BSA in hemocytes which were stained with DAPI for nucleus in blue or DiO for cytoplasmic membrane in green (A) were subjected to analysis by fluorescence microscopy. Experiment was repeated three times and representative image were shown here. The percentage of engulfed targets was obtained by multiplying the % of phagocytosing cells with the mean number of internalized beads/bacteria. Each histogram corresponds to the mean value of samples from 5–8 larvae (± S.D.). Significant differences between BmTCTP and BSA treatment are marked by asterisks (*p<0.05, **p<0.01).

To address whether the binding of TCTP to different ligands or bacteria plays a role in cellular immunity, we carried out a phagocytotic assay by injecting BmTCTP coated beads or bacteria into the hemolymph of fifth instar larvae. The hemocytes collected from the insects were subjected directly to microscopic analysis. As shown in Figure 3, endocytosed beads or bacteria were both located in the vicinity to the nucleus of hemocytes, or inside the plasma membrane as indicated by DiO staining. Compared to BSA coating, the percentage of phagocytosing cells and engulfed target was increased to a substantially greater extent in BmTCTP pretreatment, suggesting that BmTCTP promotes the phagocytosis of invading substances by hemocytes.

### BmTCTP Induces the Production of Anti-microbial Peptides through ERK Pathway

**Figure 4:**
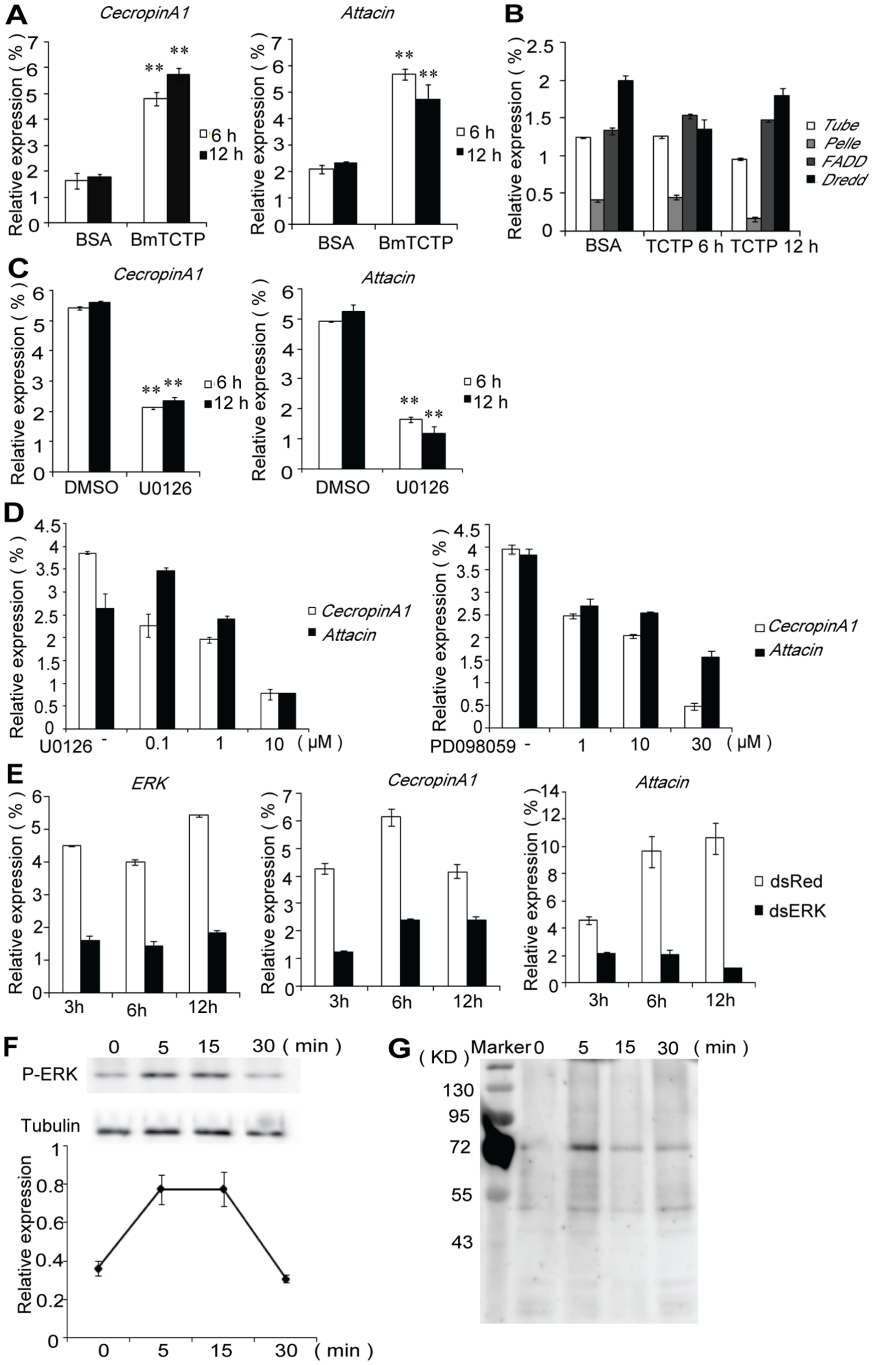
Induction of anti-microbial peptides mRNA expression and activation of ERK signaling by BmTCTP. qRT-PCR analysis of the mRNA levels of *CecropinA1* and *Attacin* (A), *Tube*, *Pelle*, *FADD* and *Dredd* (B) in BmNs cells under BmTCTP or BSA treatment. In the presence of MEK inhibitor U0126 or PD098059, the mRNA levels of *CecropinA1* and *Attacin* after BmTCTP treatment were examined at different time points (C) or at different concentrations of inhibitors (D) by qRT-PCR. E. qRT-PCR analysis of the mRNA levels of *ERK*, *CecropinA1* and *Attacin* under BmTCTP treatment at indicated time points in dsRNA-transfected cells. F. ERK phosphorylation was examined at indicated time points after BmTCTP treatment, the phosphorylation intensity was normalized to the protein level of Tubulin and annotated as mean fold increase (± S.D.) over the normalized phosphorylation intensity at the 0 min. G. Membrane protein extracted from BmNs cells under BmTCTP treatment at indicated time points was immunoblotted with anti-phosphotyrosine antibody 4G10. The mRNA levels of all target molecules measured by qRT-PCR were normalized to the internal control and represented as mean ± S.D.

TCTP is first discovered as histamine-releasing factor (HRF) and later found to be able to stimulate the production of some cytokines, such as IL-4 or IL-13 in basophils. However, its activity in insect humoral immune response is still unknown. To address this question, we examined the expression level of AMP by qRT-PCR after incubating cells with BmTCTP (Figure 4A). The expression level of *CercropinA1* and *Attacin* increased to more than 3-fold after BmTCTP induction. As a control, no increase was observed when BSA was added to the cells.

To investigate the mechanism underlying BmTCTP activity in promoting AMP production, the involvement of two canonical immune signaling pathway in insects, Toll and IMD pathway, was examined (Figure 4B). No consistent change in expression level of signaling molecules, including *Tube*, *Pelle*, *FADD* and *Dredd*, was detected. However, when mitogen-activated protein kinase kinase (MEK1/2) specific inhibitor, U0126 was applied to cells before BmTCTP treatment, the increase of *CercropinA1* and *Attacin* expression was abrogated in both time points (Figure 4C), and the inhibition was dose-dependent on the concentration of U0126 (Figure 4D), suggesting ERK signaling is required for BmTCTP-induced AMP production. We also tested another specific inhibitor for ERK activation, PD098059, and obtained similar results. To confirm the potential relevance of ERK signaling in BmTCTP activity, we used RNA interference to knock down *ERK* expression (Figure 4E). The transcriptional level of *ERK* was reduced to 30–40% by dsRNA against ERK (dsERK) when compared to dsRNA against DsRed (dsRed). Coincidently, expression of *CercropinA1* and *Attacin* in dsERK transfected cells dropped to a much lower level after BmTCTP induction. These data further supported the involvement of ERK in BmTCTP-stimulated reaction.

To monitor the signaling events induced by BmTCTP, ERK activation was determined by immunoblotting with anti-phospho-ERK antibody (Figure 4F). Because of the inability of commercial-available anti-ERK antibodies to cross react with silkworm ERK, the protein level of tubulin was used to normalize the ERK phosphorylation level at different time points. The kinetics of ERK phosphorylation showed a transient increase, with maximal level reached at approximately 5 min, persisted for about 10 min and finally decreased within 30 min. Since the ERK signaling pathway is usually coupled with certain cytoplasmic membrane protein which becomes phosphorylated on tyrosine residues when activated, we extracted the membrane protein of BmTCTP treated cells in different sampling times and immuno-blotted with anti-phosphotyrosine antibody. As a result, we found the dynamic tyrosine phosphorylation occurred in a band about 72 kDa, with the maximum signal at 5 min (Figure 4G).

## Discussion

A good understanding of insect intestinal immunity relies on thorough investigation of the physiological roles of gut protein and their interactions. Great efforts taken in genomic, physiological and biological research makes silkworm a key model organism in Lepidoptera for studies of host-pathogen interaction. A few immune-related proteins were identified in the midgut of silkworm, *Bombyx mori* by mass spectrometry or gene profiling analysis [34], [35], including β-1,3-glucan recognition protein, Aminopeptidase N, alkaline phosphatase, serine protease inhibitors, AMP and some unique protein that is believed to be important for silkworm anti-viral immunity, such as chlorophyllide A-binding protein and lipase [36], [37]. Based on their predicted functions in immune response, some of them can be categorized to be immune recognition molecules. While considering the complicated expression profile of immune-related genes upon pathogen oral infection [38], the defined immune response which is triggered by these molecules is not enough to count for the consequent defending strategies. Therefore, other approaches would be used to discover uncharacterized molecules that also participate in pathogen recognition, immune signal transduction or regulation. In search of molecules participating in certain biological process, function driven screening is a very powerful and efficient strategy that has the potential to identify completely new molecules. In this study, by phage display with pathogens as the bait, we discovered BmTCTP to be a novel multi-ligand binding molecule.

TCTP, also named IgE-dependent histamine-releasing factor (HRF), p23/p21 or fortilin, has been identified in diverse metazoa including nematodes, insects, fish, and mammals [39], [40]. TCTP from different species are characterized by homologous sequence features, including a core conserved structure similar to guanine nucleotide exchange factor (GEF) 1 for Rab protein which is involved in the secretory pathway, and two TCTP motifs which function is still unknown but is proposed to confer interaction with other molecules. It virtually binds various proteins in cells, such as P53, Bcl-xL, components of DNA damage sensing and repair, Rheb and Na,K-ATPase α subunit to take part in diverse regulatory network [41]–[45]. *BmTCTP* gene was firstly reported by Lee *et al.* [46], and its encoding protein was found to express in the midgut cavity, the midgut wall and in some fat bodies attached to the midgut in silkworm pupae by Nie *et al* [47]. In present study, we found a noticeable amount of BmTCTP lining on the membrane boundary, which was similar to the result of Amzallag *et al.* obtained in mammalian cell lines [48]. Interestingly, TCTP was also identified from *Aedes aegypti* midgut brush border membrane by proteomic approach, suggesting that it would play a potential role in interaction with exogenous molecules [49]. The absence of BmTCTP in gut fluid might be the result of its binding to chitin in peritrophic membrane, which prevents BmTCTP from passing through. The increase of BmTCTP protein level but not the transcript level (data not shown) in gut lumen was observed at the early infection stage, probably implying that the release of BmTCTP from gut epithelial cells occurs slowly unless the gut homeostasis is no longer maintained. We also found BmTCTP is present in hemolymph which might be produced by fat bodies and patrol inside the body cavity. Recently, a novel transmembrane protein has been characterized in *P. monodon* to be the binding partner of PmTCTP and probably facilitate its transport through the plasma membrane [50]. Unfortunately, no homologous molecule can be identified in silkworm.

The binding of TCTP with foreign substance was once reported by yeast two-hybrid experiment that chicken TCTP binds to Marek’s disease virus (MDV)-specific protein [51]. In our *in vitro* binding assay, we found BmTCTP could bind to different bacteria or PAMPs. This unspecific relationship between BmTCTP with multiple ligands would be explained by electrostatic interaction between them. Owing to the Lys residues aligned along the outer surface of TCTP motif and flanking a hydrophobic area to create a positively charged patch [52], TCTP would bind to highly negative charged bacterial cell wall. Such a coating of BmTCTP on the surface of bacteria leads to an enhanced ingestion of bacteria as shown in phagocytosis assay. Another multi-ligand recognition protein, cationic protein 8 (CP8), was identified in *Galleria mellonella* [53]. This protein recognizes *E. coli*, *Micrococcus luteus* and *Candida albicans* via specific binding to LPS, lipoteichoic acid (LTA) and β-1,3-glucan and acts as an opsonin. Based on this common feature, we propose BmTCTP to be a novel opsonic molecule for the phagocytosis of microorganisms. In contrast to CP8, which is predominantly present in the hemolymph but not in the protein extract from the midgut, BmTCTP seems more abundant in the gut lumen than in the hemolymph. Although our study provides evidence that BmTCTP acts as an opsonin to hemocytes, whether it is also enrolled in the phagocytosis of invading bacteria by intestinal epithelial cells is still unknown. This putative intracellular defense was rarely addressed in insect, whereas some pathogen, such as *S. marcescens*, was detected at intracellular locations, likely within autophagic vacuoles of *Drosophila* midgut epithelial cells during early infection [54]. Further investigation is needed to investigate whether TCTP is engaged in the intracellular elimination of pathogens by insect intestinal cells.

Given the notable function of TCTP in promoting allergic reaction identified in mammals, BmTCTP may also act as a pro-inflammatory factor if its evolutionary conservancy is considered. The concentration of extracellular BmTCTP in the hemolymph was estimated to be 1.0 µg/ml, so we used a concentration slightly higher than that (1.2 µg/ml) to treat BmNs cells and found the transcriptional level of *CecropinA1* and *Attacin* was significantly increased. In search of the mechanism accounting for this immune-stimulatory activity of BmTCTP, the possible involvement of Toll or IMD signaling pathway was first examined. The qRT-PCR result showed that the expression level of some essential molecules in these two pathways remained relatively stable or even slightly decreased, suggesting induction of AMP by BmTCTP is not dependent on activation of these pathways, at least not completely. However, either ERK inhibitors or RNA interference against *ERK* abrogated the activity of BmTCTP. Moreover, the dynamic ERK phosphorylation was observed after BmTCTP treatment, implicating ERK signaling pathway is activated by BmTCTP and required for the consequent AMP expression. Several studies in mammalian models also uncovered the phosphorylation of many signaling molecules, including MEK, ERK, Syk and Akt, in TCTP-bound basophils and an immunotyrosine activating motif (ITAM)-associated receptor was proposed to activate the membrane proximal signaling events [55], [56]. Although we did find tyrosine phosphorylation pattern of certain membrane protein appears in accordance with a typical ITAM-embedded signaling cascade, the receptor of BmTCTP remains to be identified.

In summary, our data demonstrated for the first time the immunological functions of TCTP in insect. It is involved in phagocytosis as a multi-ligand binding opsonin, and it also stimulates AMP production in an ERK-dependent manner. More importantly, the function-driven selection led to the identification of BmTCTP from the midgut, but the role it plays is obviously not limited to this organ. It is very likely that the results we obtained in midgut research will be of significant relevance to the integrity of the whole immune system. Overall, the study presented herein provides us a new perspective of insect immunity.
